# Social familiarity affects Diana monkey (*Cercopithecus diana diana*) alarm call responses in habitat-specific ways

**DOI:** 10.1098/rsos.150639

**Published:** 2016-02-24

**Authors:** Claudia Stephan, Klaus Zuberbühler

**Affiliations:** 1 Institute of Biology, Department of Comparative Cognition, University of Neuchâtel, Neuchâtel, Switzerland; 2 School of Psychology and Neuroscience, University of St Andrews, St Andrews, UK

**Keywords:** dear enemy effect, nasty neighbours, alarm calls, familiarity, vocal flexibility, population differences

## Abstract

Male Diana monkeys produce loud and acoustically distinct alarm calls to leopards and eagles that propagate over long distances, much beyond the immediate group. Calling is often contagious, with neighbouring males responding to each other’s calls, indicating that harem males communicate both to local group members and distant competitors. Here, we tested whether male Diana monkeys responding to each other’s alarm calls discriminated familiar from unfamiliar callers in two populations in Taï Forest (Ivory Coast) and on Tiwai Island (Sierra Leone). At both sites, we found specific acoustic markers in male alarm call responses that discriminated familiar from unfamiliar callers, but response patterns were site-specific. On Tiwai Island, males responded to familiar males’ eagle alarms with ‘standard’ eagle alarm calls, whereas unfamiliar males triggered acoustically atypical eagle alarms. The opposite was found in Taï Forest where males responded to unfamiliar males’ eagle alarm calls with ‘standard’ eagle alarms, and with atypical eagle alarms to familiar males’ calls. Moreover, only Taï, but not Tiwai, males also marked familiarity with the caller in their leopard-induced alarms. We concluded that male Diana monkeys encode not only predator type but also signaller familiarity in their alarm calls, although in population-specific ways. We explain these inter-site differences in vocal behaviour in terms of differences in predation pressure and population density. We discuss the adaptive function and implications of this behaviour for the origins of acoustic flexibility in primate communication.

## 1. Introduction

Some of the most interesting examples of complex animal behaviour come from studies on predator-specific alarm calls, including the classic studies on the vervet monkey alarm call system [1]. Here, unique acoustic structures (e.g. ‘eagle’ alarms) are typically emitted to very specific events (e.g. eagle attacks), enabling recipients to make inferences about the event witnessed by the caller. As a result, the vervet monkey alarm call system has regularly featured in discussions about the origins of human symbolic communication and language [2]. More recently, the relevance of this finding has been challenged on the grounds that context-specific communication does not require sophisticated cognitive resources, mainly because of the reliable link between signal type and external event (e.g. [3,4]).

With this study, we revisit the question of how complex primate alarm calling behaviour is, by focusing on the amount of information potentially encoded by alarm calls. In previous studies, it has been found that urgency [5], elevation [6] or mode of detection [7] can have a measurable influence on the acoustic structure of primate alarm calls, suggesting that alarm calls may encode more than predator categories. It is also known that caller identity matters, as demonstrated for example by female putty-nosed monkeys, who do not respond to call sequences of stranger males [8]. To our knowledge, no primate species has been shown, however, to encode social information, such as caller identity or familiarity, when responding to each other’s alarm calls.

There is good evidence that animals can categorize their social worlds according to functional classes, an adaptive ability that facilitates information processing (e.g. [9,10]). For example, categorization of familiar and unfamiliar conspecifics goes beyond an individual’s physical features and takes into account the previous history of encounters (e.g. [11,12]). This is important in territory defence (e.g. [13,14]), group functioning [15,16] and reproductive contexts [17,18]. This discriminative ability has been demonstrated in a wide range of species and across modalities (olfactory (e.g. [19,20]), visual (e.g. [21,22]) and acoustic (e.g. [15,23,24])). In group-living primates, familiarity judgements are of especially high relevance because most species live in socially stable clusters surrounded by neighbouring groups (e.g. wild chimpanzees [24]). At the same time, group members are regularly confronted with unfamiliar individuals due to migration, which can have considerable fitness implications for group members [25].

**Figure 1.**
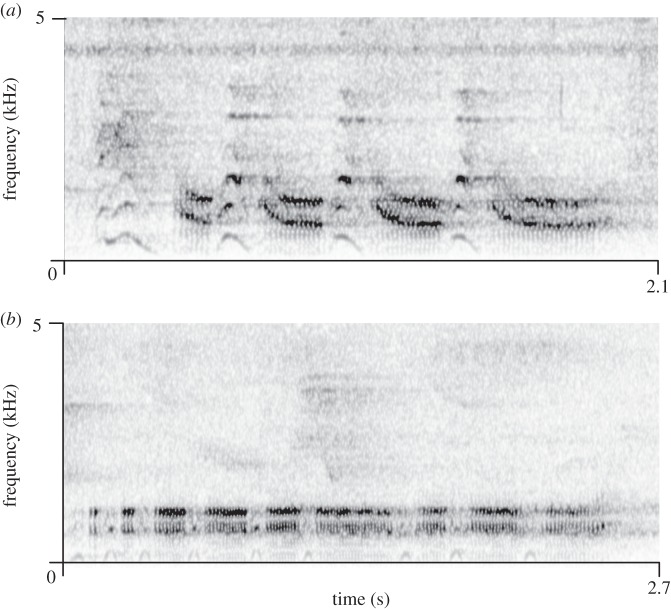
Spectrograms of examples of (*a*) male responses to leopard playback (Taï) and (*b*) male responses to eagle-related playback.

In our study, we focused on Diana monkeys, a typical forest guenon species that lives in groups with one reproductive male, several adult females and their offspring [26,27]. Males regularly give acoustically distinct loud alarm calls in response to leopards (*Panthera pardus*) and eagles (*Stephanoaetus coronatus*) and a third type of loud alarm call to general disturbances, which resembles the leopard alarm calls in its acoustic structure but is organized in longer sequences [28]. The call units given to leopards and general disturbances are characterized by a prominent decline in frequency at call onset (frequency transitions; figure 1), which is absent in eagle alarm calls.

Remarkably, male Diana monkey alarm call sequences travel over long distances in dense forest habitat (more than 800 m; K.Z. 1996, unpublished data), suggesting that they serve an additional function in male–male competition and resource defence [28,29]. Female alarm calls are much softer, suggesting that they mainly serve in within group communication [30]. Listeners respond to male and female predator-specific alarm calls as if encountering the corresponding predator, which led to the assumption that these alarm calls are highly context-specific with a rigidly conserved acoustic structure (e.g. [28]).

In previous research, we have shown that male and female Diana monkeys differ in their alarm calling behaviour in habitat-specific ways. In Taï Forest, Ivory Coast, males consistently produce all three alarm call types in event-specific ways (eagle, leopard and general disturbance), whereas on Tiwai Island, Sierra Leone—a habitat characterized by long-term absence of leopards [31]—males only distinguish between eagles on the one hand and leopards and general disturbances on the other hand. Females also differ in their vocal responses to leopard-related events in habitat-specific ways (in terms of call rates, latencies but not acoustic structure [32]). Crowned eagles are present in both habitats and male alarm call responses to them are similar across both habitats. In particular, male alarm calls do not show any frequency transitions at call onset (figure 1) at either site, and call sequences are uniformly long. Diana monkey groups at both sites used to be part of the same continuous forest population with no differences in social organization [27,33], territory size [34,35] and other socio-ecological parameters.

In this study, we simulated the presence of familiar and unfamiliar males to wild Diana monkeys in Taï Forest (Ivory Coast) and Tiwai Island (Sierra Leone) by playing back previously recorded alarm calls to leopard and eagle playbacks. Given the social organization of Diana monkeys, we expected both populations to discriminate conspecifics by their vocalizations alone on the basis of familiarity. We were especially interested in whether Diana monkey males were able to acoustically mark familiarity and predator type within the same vocal signal. If males also encoded social classification in their own responses, we predicted similar effects in both populations for eagle-related alarms. Given previously found population differences in responses to leopard playbacks, we expected males to extract different information regarding the context-specificity of these alarms in Taï and on Tiwai. Hence, if the discrimination of familiar and unfamiliar males is only relevant in specific predatory contexts (i.e. to infer the urgency of predation attempts by leopards), we hypothesized Taï males to show this assessment in alarms but not Tiwai males. However, if this socially related information were relevant on its own, categorization should be encoded in both habitats also to alarms induced by leopard playbacks.

## 2. Material and methods

### 2.1 Subjects and playback stimuli

We tested different groups of Diana monkeys in Taï forest (*N*=20, August to December 2013) and on Tiwai Island (*N*=23, January to March 2014) with playbacks of familiar and unfamiliar male Diana monkey alarm calls to leopards and eagles. Playback stimuli were obtained by simulating the presence of a crowned eagle or a leopard by playing back their typical vocalizations (*N*_leopard growls_=3; *N*_eagle shrieks_=3) to different males. We then used these responses as playback stimuli to elicit alarm call responses from our target subjects [6,31,32]. At both sites, playbacks could be from a familiar male, defined as an alarm call response recorded from a neighbouring group’s male of the same habitat, or an unfamiliar male. Playbacks of unfamiliar males’ calls were recorded from a non-neighbouring group’s male more than 2 km from the target male’s current location, but from the same habitat. From the obtained recordings, we then created standardized sequences consisting of three call sequences separated by 5 s of silence, assigned to four different conditions, namely alarms to eagle and leopard from familiar and unfamiliar males, respectively; table 1. All playback stimuli were recorded in the subjects’ respective habitats and used in both the familiar and unfamiliar condition, to rule out that callers could categorize the stimulus by cues other than familiarity.

**Table 1.** Number of different males that served as playback stimuli for each condition in Taï and on Tiwai (*N*_stimuli_) and number of groups/males that were tested (*N*_groups_) in each condition (leopard familiar: neighbouring male’s alarm calls to leopard playback; leopard unfamiliar: unknown male’s alarm calls to leopard playback; eagle familiar: neighbouring male’s alarm calls to eagle playback; eagle unfamiliar: unknown male’s alarm calls to eagle playback).

	Taï		Tiwai	
	*N*_stimuli_	*N*_groups_	*N*_stimuli_	*N*_groups_
leopard familiar	7	9	7	8
leopard unfamiliar	6	8	8	9
eagle familiar	6	6	6	8
eagle unfamiliar	8	8	8	10

We only used good quality recordings made at an adequate recording distance with no overlap between the male alarm calls and other individuals’ calls (females or other species). If a neighbouring male responded during the trial, we did not use the recording for further playbacks.

### 2.2 Experimental procedure

Each subject male was only tested once per condition although not all males were tested in each condition. The experimenter first located the group by auditory cues before silently approaching the tree occupied by most group members to a distance of about 10 m. The group was then monitored for about 15 min without being detected. After the playback and recording equipment was positioned at the same place, the baseline vocal behaviour of the group was recorded for at least 3 min before the playback stimulus was broadcasted. The male’s vocal response was recorded, together with all other group members’ responses, until all of them stopped alarm calling or for at least 10 min. For each trial, we estimated the group size, identified all other monkey species present and noted their vocal responses. Trials during which disturbing events occurred, such as the sudden appearance of a large mammal (*N*=2), the sudden alarm calling of another group (*N*=6) or any natural predator attacks (*N*=1) were excluded from further analyses. We also excluded trials with insufficient recording quality due to technical problems (*N*=5) or in which the target male’s response overlapped with female calls or other species’ calls (*N*=23). Finally, we excluded all trials in which another monkey species started alarm calling before the focal Diana monkey group (*N*=15). We chose this precautionary measure to exclude that Diana monkeys simply responded to the hetero-specific alarm calls rather than to the playback stimulus *per se*. In total, we included responses from 20 males in Taï and from 23 males on Tiwai (number of males equal the number of different groups due to the social structure of Diana monkeys; see table 1 for the number of tested males in each condition).

All stimuli were broadcasted using an Apple iPod nano digital player connected to an AER alpha speaker amplifier. Vocal responses were recorded with a Sennheiser ME67 directional microphone and a Marantz PMD 660 solid-state recorder (44.1 kHz sampling rate, 16 bits amplitude resolution and stored in wav format). We never broadcasted the same stimulus within a radius of 500 m for at least two weeks to prevent eventual habituation effects.

### 2.3 Data analysis and statistics

We carried out acoustic analyses for both habitats across the four conditions on single calls (‘call-related parameters’) and described the call sequences in terms of length and occurrence of frequency transitions at call onset (‘sequence-related parameters’). We also compared the vocal responses of males in general terms (‘response-related parameters’) as the proportion of vocal responses per valid trials (*response rate*), the latency to respond, the proportion of different alarm call types and the number of call sequences per response.

We extracted *N*=3002 calls for Tiwai and *N*=1908 calls for Taï, which were normally assembled into sequences (figure 1). We then compared male responses to familiar and unfamiliar alarms separately for Tiwai and Taï. As long as not indicated otherwise, statistical analyses were conducted using R v. 13.0.1.

#### 2.3.1 Call-related parameters

At the level of individual calls, we analysed the presence of frequency transitions at the onset of calls [32] as well as several other frequency-related and temporal parameters. For this purpose, we used the PRAAT DSP package (settings: time step: 0.03 s; expected F0 frequency range: 500–2000 Hz), with an automatic logger in an output file to extract the following variables: (a) mean fundamental frequency (mean_F0; Hz), (b) maximum and minimum fundamental frequency (max_F0 and min_F0; Hz), (c) range of fundamental frequency (range_F0; Hz), (d) fundamental frequency at the beginning and end of a call (start_F0, end_F0; Hz). Additionally, the following temporal parameters were extracted: (e) time of maximum and minimum fundamental frequency (tF0_max and tF0_min; s) and (f) time at the beginning and end of fundamental frequency (tF0_start and tF0_end; s). From the latter, the duration of calls was calculated (call length; s), resulting in 11 parameters for each call that entered the subsequent analysis. We then conducted a principal component analysis (PCA) to reduce the original variable set to independent components that did not correlate with each other. Before independent variables were created, the original dataset was weighted according to the variable ‘group’ to control for unequal contribution of groups in each condition. Components with eigenvalues of at least 1 were extracted and a varimax-rotated correlation method was used. This analysis was conducted by means of SPSS v. 19.

We found that, for Tiwai, one variable (end_F0) had a communality value below 0.5 and two variables had high loadings (more than 0.4) on more than one component (start_F0, tF0_start), which is why we decided to eliminate three of our originally measured variables [36], resulting in eight variables with simple structure (table 2*a*). For Taï, two variables showed complex structure and were eliminated (mean_F0, tF0_start), resulting in nine variables that entered analysis (table 2*b*). A second PCA with this reduced set of variables resulted in three principal components (PC) for each habitat.

**Table 2.** Rotated component matrix for (*a*) Tiwai and (*b*) Taï. Loadings of original variables on the different components are presented. Loadings higher than 0.4 are highlighted in italic.

	component		
	1	2	3
(*a*)			
mean_F0	0.085	*0*.*888*	0.276
max_F0	0.187	*0*.*967*	-0.018
min_F0	-0.057	0.023	*0*.*975*
range_F0	0.195	*0*.*951*	-0.188
tF0_max	*0*.*834*	0.017	-0.111
tF0_min	*0*.*732*	0.168	0.201
tF0_end	*0*.*951*	0.189	-0.065
call_length	*0*.*923*	0.194	-0.123
(*b*)			
max_F0	0.122	*0*.*957*	0.114
min_F0	-0.036	-0.073	*0*.*922*
range_F0	0.129	*0*.*960*	-0.148
start_F0	-0.096	*0*.*757*	0.280
end_F0	-0.079	0.397	*0*.*714*
tF0_max	*0*.*878*	-0.141	-0.018
tF0_min	*0*.*665*	0.020	0.118
tF0_end	*0*.*957*	0.086	-0.161
call_length	*0*.*841*	0.171	-0.257

The so condensed variables were then compared within habitats among all conditions using Kruskal–Wallis tests and, in the case of overall differences, with pairwise comparisons of conditions for each extracted component using two-tailed Mann–Whitney *U*-tests. Significance levels were adjusted with a Bonferroni correction (*α*<0.0125) to account for multiple testing.

#### 2.3.2 Sequence-related parameters

We compared the sequence composition of male calls in both habitats across playback conditions. Here, we analysed the number of single calls within each call series using Kruskal–Wallis tests. If significant differences emerged between the four conditions, we conducted pairwise comparisons using two-tailed Mann–Whitney *U*-tests and adjusted the *α* level appropriately using a Bonferroni correction (*α*<0.0125). Finally, for each condition, we determined the proportion of calls without frequency transitions per calling sequence.

#### 2.3.3 Response-related parameters

To assess differences in the potential benefit of responding vocally to conspecific alarm calls, we compared the response rates across all playback conditions by means of a *χ*^2^-test. Similarly, we analysed the latency to respond as another indication of perceived urgency of displayed alarm calls by means of Kruskal–Wallis and Mann–Whitney *U*-tests (including a Bonferroni correction at *α*<0.0125). Both parameters were supposed to mirror the cost–benefit ratio for a receiver to signal himself, assuming that fast alarm calling hints to a high urgency to respond with lower costs out of, for example, revealing one’s own position, and no alarm calling at the other end of the continuum indicating low benefits of calling and/or higher costs of advertising one’s presence and position. The number of call sequences within a response was compared between familiar and unfamiliar calls to eagles and leopards, respectively, using Kruskal–Wallis and Mann–Whitney *U*-tests (including a Bonferroni correction at *α*<0.0125).

## 3. Results

### 3.1 Call-related parameters

To eagle alarm calls, responses showed frequency transitions as a function of signaller familiarity in habitat-specific ways. In particular, in response to *familiar* males’ eagle alarm calls, *Tiwai* males responded with typical eagle alarms at the beginning of their call sequences (no frequency transitions; eight of eight responses), while to *unfamiliar* males, they started sequences with calls that showed frequency transitions (10 of 10 responses), similar to leopard or general alarm calls. In *Taï*, the pattern was reversed in that males immediately started sequences with calls that showed frequency transitions to familiar males’ eagle alarm calls (six of six responses), and with typical eagle alarm calls to *unfamiliar* males (no frequency transitions; eight of eight responses).

At both sites, subsequent calls in all sequences mostly showed calls with frequency transitions (see below: *sequence-related parameters*). To analyse whether calls to eagle alarms that showed frequency transitions still encode any context-specific information about the event that triggered them (eagle presence and signaller familiarity), we compared PC across all playback conditions for Taï and for Tiwai. Component 1 could be assigned to the temporal parameters of the calls, whereas components 2 and 3 corresponded to pitch-related variables in both habitats (table 2).

Specifically, *for Tiwai*, non-parametric *post hoc* comparisons between playback conditions revealed overall differences in the first and second principal component (PC1: Kruskal–Wallis test: *χ*^2^=38.3, d.f.=3, *p*<0.001, total variance explained: 48%; PC2: Kruskal–Wallis test: *χ*^2^=32.9, d.f.=3, *p*<0.001, total variance explained: 25%; see table 3*a* for means of single variables) but not in the third (PC3: Kruskal–Wallis test: *χ*^2^=7.3, d.f.=3, *p*=0.064, total variance explained: 14%). For component 1 (temporal features), calls to *unfamiliar eagle alarms* (atypical eagle alarms with frequency transitions) were shorter and reached maximum, minimum and end fundamental frequency earlier than to any other playback condition. For component 2 (pitch-related variables), responses to *unfamiliar eagle alarms* consistently showed lower mean and maximum fundamental frequencies and a smaller F0 range than responses to any other playback condition (table 4*a*). By this, even showing frequency transitions that are usually characterizing alarms to leopards and general disturbances, responses to unfamiliar eagle alarms were reliably different from other responses.

**Table 3.** Mean and standard deviation of single variables that loaded on PC showing significant differences between playback conditions for (*a*) Tiwai (PC1: time of maximum and minimum fundamental frequency (tF0_max and tF0_min; s), time at the end of fundamental frequency (tF0_end; s) and call length (s); PC2: mean fundamental frequency (mean_F0; Hz), maximum fundamental frequency (max_F0; Hz) and range of fundamental frequency (range_F0; Hz)) and (*b*) Taï (PC1: tF0_max, tF0_min, tF0_end and call length, PC3: minimum fundamental frequency (min_F0; Hz) and fundamental frequency at the end of a call (end_F0; Hz)).

	leopard familiar (*N*=8) mean±s.d.	eagle familiar (*N*=8) mean±s.d.	leopard unfamiliar (*N*=9) mean±s.d.	eagle unfamiliar (*N*=10) mean±s.d.
(*a*)				
tF0_max	0.8±0.06	0.9±0.04	0.81±0.06	0.63±0.04
tF0_min	0.6±0.05	0.86±0.07	0.58±0.05	0.39±0.02
tF0_end	1.63±0.09	1.75±0.16	1.35±0.01	0.99±0.08
call_length	1.53±0.09	1.6±0.08	1.22±0.01	0.88±0.03
mean_F0	831±101	869±102	914±86	774±56
max_F0	1368±104	1350±126	1436±122	1197±80
range_F0	837±78	790±59	902±85	673±55

	leopard familiar (*N*=9) mean±s.d.	eagle familiar (*N*=6) mean±s.d.	leopard unfamiliar (*N*=8) mean±s.d.	eagle unfamiliar (*N*=8) mean±s.d.
(*b*)				
tF0_max	0.49±0.05	0.96±0.04	0.55±0.06	1.07±0.08
tF0_min	0.55±0.06	1.42±0.09	0.56±0.05	1.13±0.07
tF0_end	1.03±0.05	1.99±0.08	1.34±0.08	1.98±0.04
call_length	0.94±0.08	1.82±0.08	1.24±0.1	1.66±0.5
min_F0	576±78	530±30	565±37	566±105
end_F0	928±101	789±121	851±67	848±91

**Table 4.** Pairwise comparisons of PC extracted from response calls to different playback conditions for (*a*) Tiwai and (*b*) Taï (leopard familiar: neighbouring male’s alarm calls to leopard playback; leopard unfamiliar: unknown male’s alarm calls to leopard playback; eagle familiar: neighbouring male’s alarm calls to eagle playback; eagle unfamiliar: unknown male’s alarm calls to eagle playback). Significant *p*-values are highlighted in italic.

	component			
	PC1 (tF0_max, tF0_min, tF0_end, call length)		PC2 (mean_F0, max_F0, range_F0)	
pairwise comparison	*U*	*p*	*U*	*p*
(*a*)				
leopard familiar: leopard unfamiliar	2089	0.231	2022	0.142
leopard familiar: eagle familiar	646	0.653	647	0.661
leopard familiar: eagle unfamiliar	1238	<*0*.*001*	1567	*0*.*001*
leopard unfamiliar: eagle familiar	1704	0.727	1499	0.18
leopard unfamiliar: eagle unfamiliar	3767	<*0*.*001*	3663	<*0*.*001*
eagle familiar: eagle unfamiliar	1019	<*0*.*001*	1179	*0*.*003*

	component			
	PC1 (tF0_max, tF0_min, tF0_end, call length)		PC3 (min_F0, end_F0)	
pairwise comparison	*U*	*p*	*U*	*p*
(*b*)				
leopard familiar: leopard unfamiliar	5887	0.192	4415	<*0*.*001*
leopard familiar: eagle familiar	943	<*0*.*001*	1805	<*0*.*001*
leopard familiar: eagle unfamiliar	3366	<*0*.*001*	4408	<*0*.*001*
leopard unfamiliar: eagle familiar	725	<*0*.*001*	1703	0.683
leopard unfamiliar: eagle unfamiliar	1934	<*0*.*001*	2775	0.287
eagle familiar: eagle unfamiliar	1426	0.18	1353	0.082

*For Taï*, overall differences between playback conditions were found for the first and third principal component (PC1: Kruskal–Wallis test: *χ*^2^=71.1, d.f.=3, *p*<0.001, total variance explained: 35%; PC3: Kruskal–Wallis test: *χ*^2^=34.7, d.f.=3, *p*<0.001, total variance explained: 15%; see table 3*b* for means of single variables) but not for the second (Kruskal–Wallis test: *χ*^2^=5.3, d.f.=3, *p*=0.154, total variance explained: 29%). Pairwise comparisons revealed that males’ calls that were emitted to *leopard-elicited conspecific alarms* were shorter and showed earlier maximum, minimum and end fundamental frequency than compared with eagle-elicited conspecific alarms (component 1). Regarding the pitch-related component (composed in Taï of the minimum fundamental frequency and the fundamental frequency at the end of single calls, component 3), males uttered calls to *familiar leopard alarms* that showed higher minimum fundamental frequencies and higher fundamental frequencies at the end than to any other playback type (table 4*b*).

### 3.2 Sequence-related parameters

*For Tiwai*, the number of calls assembled into sequences varied significantly as a function of playback condition but this was not the case for Taï (Kruskal–Wallis test: Tiwai: *χ*^2^=8.4, d.f.=3, *p*=0.039; Taï: *χ*^2^=4.7, d.f.=3, *p*=0.196). Pairwise comparisons revealed that Tiwai males consistently uttered longer call sequences to *familiar eagle males* than to any other playback condition, although after Bonferroni correction, this effect remained significant only between familiar and unfamiliar males’ eagle responses (two-tailed Mann–Whitney *U*-test: eagle familiar (*N*=48, median=9, range: 2.5–23.5)–eagle unfamiliar (*N*=313, median=3.5, range: 2–5): *U*=68.5, *p*=0.011; eagle familiar–leopard familiar (*N*=73, median=4, range: 3–10.5): *U*=14.5, *p*=0.043; eagle familiar–leopard unfamiliar (*N*=138, median=4, range: 2–9): *U*=15.5, *p*=0.048).

Calls without frequency transitions, i.e. typical eagle alarms, were reliably uttered in early responses to eagle alarms of familiar males (Tiwai) or unfamiliar males (Taï). However, at both sites, males produced calls with frequency transitions during later parts of the sequences although this varied between study sites (Tiwai: proportion of non-frequency-transition calls to familiar eagle alarms: median=56%, range 29–100%; Taï: proportion of non-frequency-transition calls to unfamiliar eagle alarms: median=26%, range 11–100%). In contrast, no target male ever started by producing calls with frequency transitions to then switch to non-frequency-transition calls. To leopard-induced stimuli, target males always started with calls showing frequency transitions.

### 3.3 Response-related parameters

**Figure 2.**
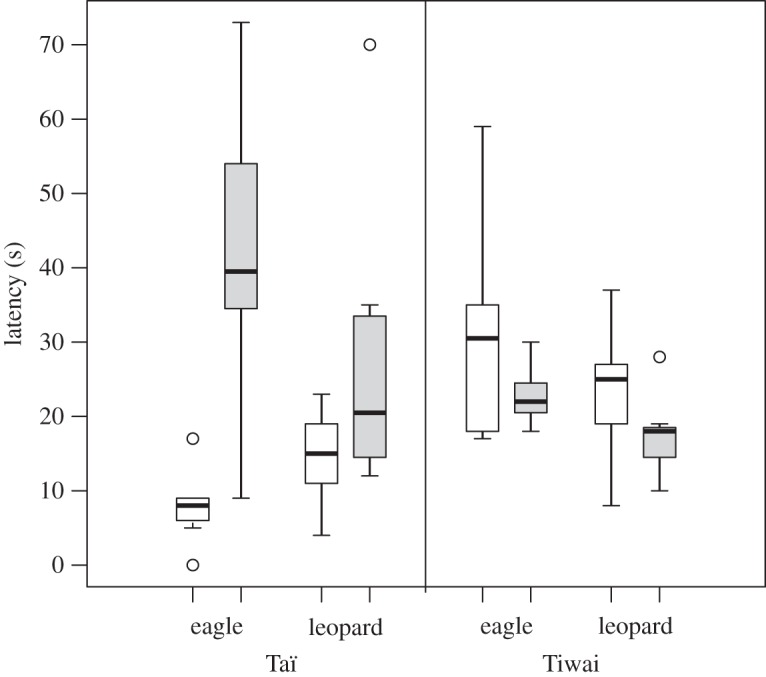
*Latency to respond* to playbacks of familiar (white boxes) and unfamiliar (grey boxes) males. The bottom of the box indicates the first, and the top of the box the third quartile. The horizontal line within the box represents the median. Whiskers include values that amount to 1.5 times the height of the box. Circles indicate outliers that do not fall in the inner fences (whiskers).

Response rates did not differ across playback conditions in both habitats (Taï: *χ*^2^=1.7, d.f.=3, *p*=0.63; Tiwai: *χ*^2^=2.6, d.f.=3, *p*=0.46). We analysed the latency to respond for both habitats (figure 2) and found no significant effect of playback condition for Tiwai (Kruskal–Wallis test: *χ*^2^=6, d.f.=3, *p*=0.111), but a significant effect for *Taï* (*χ*^2^=16.8, d.f.=3, *p*<0.001). Specifically, responses to familiar males’ eagle alarm calls started faster than to unfamiliar males’ alarm calls (two-tailed Mann–Whitney *U*-test: eagle familiar (*N*=6, median=7.5 s, range: 1–17 s)–eagle unfamiliar (*N*=8, median=43 s, range: 9–73 s): *U*=54, *p*=0.002; eagle familiar–leopard unfamiliar (*N*=8, median=20.5 s, range: 12–70 s): *U*=53.5, *p*=0.003). Males also responded faster to familiar alarms to leopards than to unfamiliar alarms to eagles but not to unfamiliar calls to leopards (leopard familiar (*N*=9, median=15 s, range: 4–23 s)–eagle unfamiliar: *U*=6, *p*=0.006; leopard familiar–leopard unfamiliar: *U*=15.5, *p*=0.082). We found no differences in the latency to respond between familiar alarm calls (leopard familiar–eagle familiar: *U*=42, *p*=0.104), nor between unfamiliar alarm calls (pairwise comparison: leopard unfamiliar–eagle unfamiliar: *U*=48.5, *p*=0.083).

**Figure 3.**
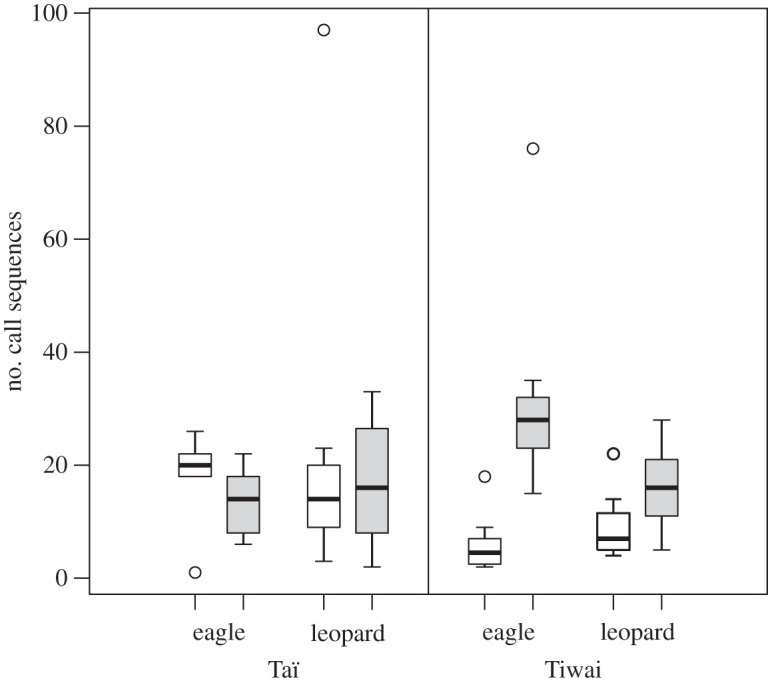
*Number of call sequences* to playbacks of familiar (white boxes) and unfamiliar (grey boxes) males. The bottom of the box indicates the first, and the top of the box the third quartile. The horizontal line within the box represents the median. Whiskers include values that amount to 1.5 times the height of the box. Circles indicate outliers that do not fall in the inner fences (whiskers).

The number of call sequences that were uttered by males varied significantly with playback condition on *Tiwai* (Kruskal–Wallis test: *χ*^2^=22.3, d.f.=3, *p*<0.001) but not in Taï (*χ*^2^=1.1, d.f.=3, *p*=0.78; figure 3). More specifically, we found males on Tiwai to emit significantly more call sequences towards unfamiliar males’ responses to eagles than to all other playbacks (two-tailed Mann–Whitney *U*-test: leopard familiar (*N*=8, median=7, range: 4–22)–eagle unfamiliar (*N*=10, median=28, range: 15–76): *U*=1.5, *p*<0.001; leopard unfamiliar (*N*=9, median=16, range: 5–28)–eagle unfamiliar: *U*=81, *p*=0.003; eagle familiar (*N*=8, median=4.5, range: 2–18)–eagle unfamiliar: *U*=79, *p*<0.001). Furthermore, males’ responses consisted of more call series towards unfamiliar males’ responses to leopards than compared with familiar males’ responses to eagles (two-tailed Mann–Whitney *U*-test: leopard unfamiliar–eagle familiar: *U*=63, *p*=0.009). No differences in the number of call sequences were found between responses to familiar alarms (two-tailed Mann–Whitney *U*-test: leopard familiar–eagle familiar: *U*=46.5, *p*=0.126) and between alarms to leopard playbacks (two-tailed Mann–Whitney *U*-test: leopard familiar–leopard unfamiliar: *U*=17.5, *p*=0.074).

## 4. Discussion

Our results show that free-living male Diana monkeys distinguish not only the external event encoded in conspecific alarm calls but also whether or not they are familiar with the caller. Moreover, familiarity was acoustically encoded in their alarm calls although this occurred in a habitat-specific way. To our knowledge, this is the first empirical evidence of primate loud alarm calls acoustically encoding information from two different contexts, namely information about the type of event and the familiarity of the information provider, suggesting that the same signal functions to transmit two types of information. The discrimination of others’ alarms on the basis of familiarity with the signaller seems to be a species-general cognitive capacity in Diana monkeys. Similar evidence from South African vervet monkeys suggests that monkeys respond more strongly to barks (emitted to leopards and during intra-specific agonistic interactions), if the calls originate from unfamiliar than familiar males [37].

The eliciting event underlying others’ alarms had a crucial impact on whether social familiarity was encoded in responses or not. In Taï, where leopards and eagles represent considerable threats to monkeys, male Diana monkeys indicate their inferences about the eliciting event and the familiarity of the signaller to both leopard and eagle alarms in their own responses. In contrast, on Tiwai, where eagles but not leopards are present, familiarity with the signaller was seen in responses to eagle- but not to leopard-induced alarm calls, which in this population resemble alarms to general threats. Hence, on Tiwai, males’ familiarity with the signaller does not seem to be important in responses to leopard/general disturbances.

Another surprising finding in our study was that the same acoustic feature (frequency transitions at call onset) was used in opposing ways to mark familiarity of eagle alarms in our two study populations, suggesting that learning is needed for recipients to benefit from this information [38]. How can these site-specific vocal responses to strangers be explained? Eagles usually attack in the upper canopy, predominantly occupied by Diana monkeys, giving individuals little time to react appropriately. The prediction then is that callers should produce typical eagle alarms if predation is likely to be imminent, instead of prioritizing other issues relating to male–male competition [39]. Although eagles are abundant in both habitats [40], there may be site differences in their respective hunting behaviour. Crowned eagles on Tiwai do not appear to abandon a hunt after detection by Diana monkeys (C.S. 2007, 2014, unpublished data). Instead, we have observed Tiwai eagles to repeatedly attack the same group within minutes, sometimes also hunting in pairs and approaching from different directions. Consequently, hearing another male’s eagle alarms on Tiwai may well be interpreted as high levels of predation risk in the direct vicinity [6]. This inference might either result from directly assessing stored information about eagles’ hunting behaviour or because neighbouring groups have been previously associated with higher degrees of reliability on the basis of statistic regularity, thus strengthening the associative link between familiar alarm calls and predator presence. In Taï, eagles appear to rely more on stealth and ambush and are more likely to abandon predation attempts once detected [40]. Accordingly, hearing neighbouring groups’ eagle alarms might be a reliable indicator of a low predation risk, as the attack has already happened and is unlikely to be followed by further attempts on the caller’s own group.

Another hypothesis is related to population differences in intergroup relations. On Tiwai, poaching by humans is frequent and primate abundance is probably much lower than in the relatively protected study area in Taï forest (Tiwai: 30–43 Ind km^−2^ [34]; Taï: 48–70 Ind km^−2^ [41,42] (on the basis of [43])). Accordingly, Tiwai males may be less challenged by territorial disputes and, in accordance with this, we found that group encounters on Tiwai were more likely to result in peaceful retreats than aggressive interactions compared with Taï [42]. Hence, although Tiwai males most likely recognize their neighbours, competition over resources is probably much lower compared with Taï, and we often observed neighbouring groups sharing the same areas for foraging. Hence, hearing a neighbouring male from a resource also used by the target group may not trigger territorial behaviour (‘dear enemy effect’ [44]), whereas hearing a stranger male is likely to do so.

In contrast, our study area in Taï National Park has very high primate densities, suggesting that the habitat is fully satiated with no free territories available. As a result, Taï males are likely to experience strong intergroup competition leading to a highly aggressive attitude towards familiar males and their groups, who are a constant threat to their resources (‘nasty neighbour effect’), an effect also observed in other species [45–47]. Stranger males, in contrast, are likely to be solitary males roving the forest, but these are not relevant as competitors for resources. Nasty neighbour effects tend to be relevant in social species where resident individuals outnumber potential intruders (e.g. [3]).

Thus, to understand why males showed population-level differences in whether they prioritized the social or the predation aspect in their calling responses, it may be necessary to consider the combination of low eagle/high neighbour threat in Taï and high eagle/low neighbour threat on Tiwai. As a consequence, Taï males may prioritize the neighbour aspect, while Tiwai males may prioritize the predatory threat.

Regarding responses to leopard-induced alarms, we did not find any familiarity-related differences on Tiwai (apart from a trend in the number of call sequences). As mentioned earlier, Tiwai males have no experience with leopards and respond to them as a general threat, similar to falling trees, large terrestrial mammals or neighbouring groups. The fact that caller familiarity is not marked in Tiwai males’ responses to others’ leopard alarms suggests that there is no general ‘dear enemy effect’ in all alarm calling, but that this is context-dependent. This is in line with results from Taï, where leopards are a real threat and where we found frequency-related differences in male responses to familiar and unfamiliar males’ leopard alarms.

We found no clear differences in the response rates across playback conditions and only slight differences in response latencies in Taï, suggesting that the perceived urgency was comparable across conditions. Whether males’ responses were driven by mental representations of the simulated event, which contained information about predator type and caller identity (e.g. [48–51]), or whether males were put into particular states of arousal (e.g. [52,53]) cannot be decided with our study and requires further research. However, the fact that familiarity with the caller and predator type was consistently encoded in the alarm calls at both sites suggests that receivers can extract both types of information, irrespective of the psychological mechanisms driving the caller.

In sum, we here show that Diana monkey responses to conspecific alarm calls are surprisingly flexible in that recipients not only categorize the calls of others in terms of predator class but also in terms of who has produced the call. Familiarity with the caller was a key factor in both populations, but males appeared to differ whether they perceived this as an additional threat. A combination of differences in predation pressure by crowned eagles and leopards and intergroup competition is likely to explain the differences in calling behaviour. On Tiwai Island, the threat of eagle predation is potentially overriding any potential intra-species competition, whereas in Taï the pattern may be going the other way. Intergroup competition is almost certainly higher in Taï, to the effect that familiar callers are classified as ‘nasty neighbours’ (Taï), as opposed to ‘dear enemies’ (Tiwai). Variable assessments of neighbouring threats have also been described in other species, such as in seasonal changes of aggressive behaviour towards neighbours in songbirds [47].

Overall, our results suggest that Diana monkeys are able to integrate and communicate both predator and neighbour information by switching priorities in terms of which information is more emphasized in their calls and that they do so in habitat-specific ways. These socially driven modifications in acoustic features add evidence for flexibility in an otherwise inflexible calling system and complement previous results on structural differences in call sequences.

## Data accessibility

The data used in this study are available from the Dryad Digital Repository: http://dx.doi.org/10.5061/dryad.6s0s8.
